# Neutropenic Enterocolitis Secondary to Sulfasalazine in a Woman With Psoriatic Arthritis

**DOI:** 10.7759/cureus.8576

**Published:** 2020-06-12

**Authors:** Sylvester Homsy, Ahmed Elfiky, Mohammad Abureesh, Danial Daneshvar, Alexander Bershadskiy

**Affiliations:** 1 Internal Medicine, Staten Island University Hospital - Northwell Health, Staten Island, USA; 2 Hematology and Oncology, Staten Island University Hospital, Staten Island, USA

**Keywords:** sulfasalazine, neutropenic enterocolitis, typhlitis, neutropenia, psoriatic arthritis

## Abstract

Neutropenic enterocolitis (NE) also known as typhlitis is a serious condition that has been described in immunosuppressed hosts including patients with leukemia, HIV and in patients on chemotherapy. We present the first case of female on sulfasalazine for psoriatic arthritis, otherwise healthy, who was diagnosed with NE involving the cecum and rectum. This adds up to the cases of NE diagnosed in nononcologic conditions.

A 65-year-old female with a history of psoriatic arthritis on sulfasalazine, presented to the emergency department (ED) after an episode of syncope. She was complaining of a fever and mild generalized abdominal pain. Physical exam was remarkable for peri-umbilical tenderness. Severe neutropenia and acute kidney injury were found on blood work. CT scan of the abdomen showed evidence of colitis, involving the cecum, ascending colon and rectum, which in light of neutropenia was consistent with NE. Clostridium difficile colitis was ruled out. Intravenous fluids and broad-spectrum antibiotics were initiated, and sulfasalazine was discontinued. The patient was subsequently afebrile and was out of neutropenia by day 3 without the need for granulocyte-macrophage colony-stimulating factor (GM-CSF). By day 5, the patient was pain free and was discharged.

Even though NE is primarily described in the setting of malignancies and chemotherapy, one should keep in mind that this entity can occur in people on any immunosuppressive therapy. Early discontinuation of sulfasalazine and conservative management were essential in the treatment of NE in this case. Whether neutropenia precipitates colitis or the latter causes agranulocytosis by bone marrow suppression through cytokines remains to be proved. The diagnosis of medication-related adverse reactions remains a big challenge for clinicians and therefore requires a high index of suspicion. Resolution of the symptoms can simply occur with the discontinuation of the offending drug and often does not require extensive workup or treatments that might cause harm to the patient’s health.

## Introduction

Neutropenic enterocolitis (NE), also known as typhlitis, is a serious condition that has been described in immunosuppressed hosts, including patients with leukemia, HIV and in patients on chemotherapy. It was first described by Moir and Bale in 1976 in children with leukemia, and it has also been reported to occur with the use of many chemotherapeutic agents [1,2]. Sulfasalazine, an aminosalicylic acid used mostly in ulcerative colitis, has been implicated in a fatal case of NE in a patient with rheumatoid arthritis [3]. Our patient, who has been treated with sulfasalazine for her psoriatic arthritis, was found to have NE involving the cecum and the rectum and recovered after five days of hospital stay.

## Case presentation

**Figure 1:**
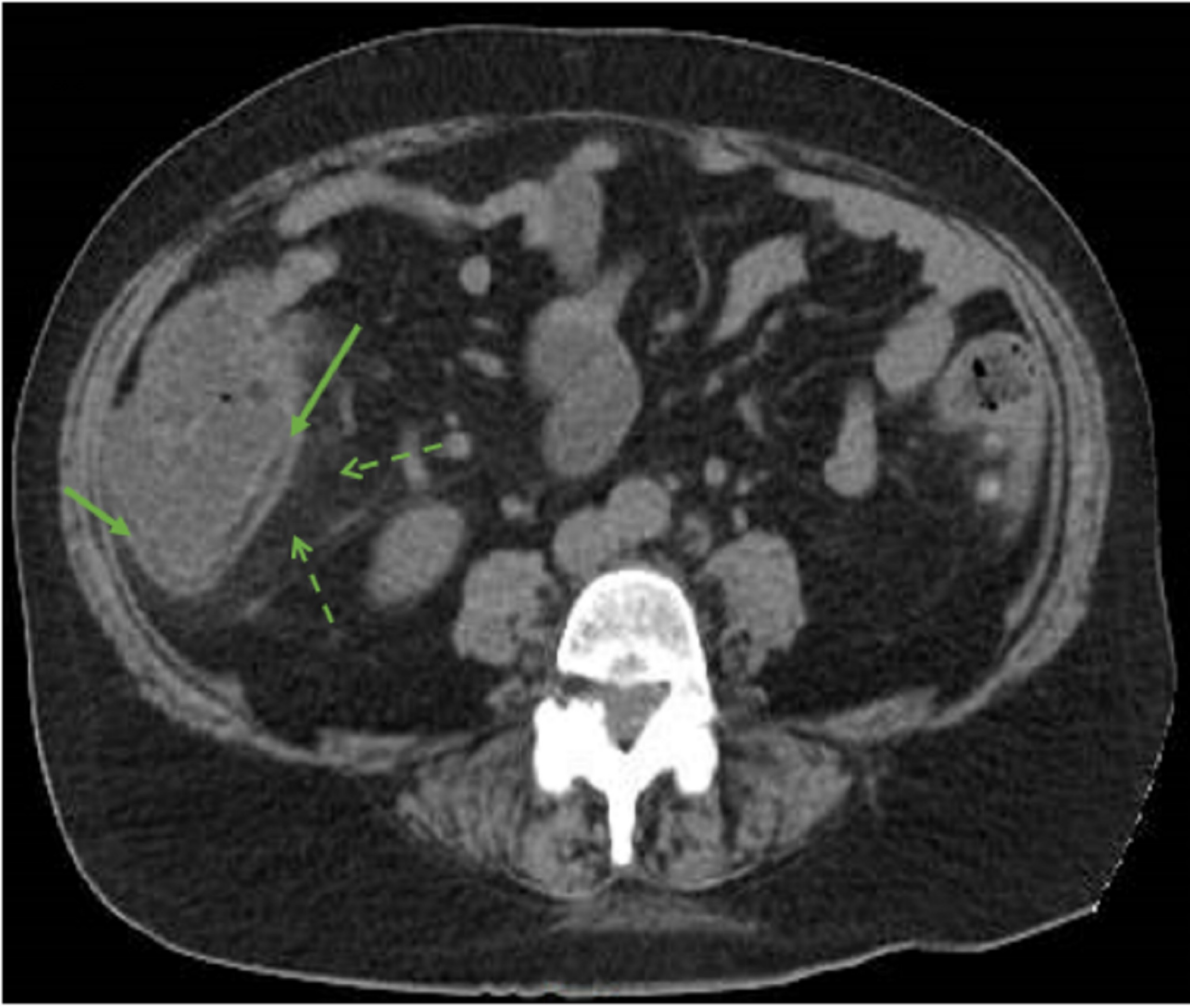
Inflammatory changes of cecum and ascending colon compatible with typhlitis. CT of the abdomen with intravenous contrast at the level of the cecum showing a circumferential wall thickening of the cecum and ascending colon (full arrows) with adjacent mesenteric fat stranding (dashed arrows) consistent with colitis.

**Figure 2:**
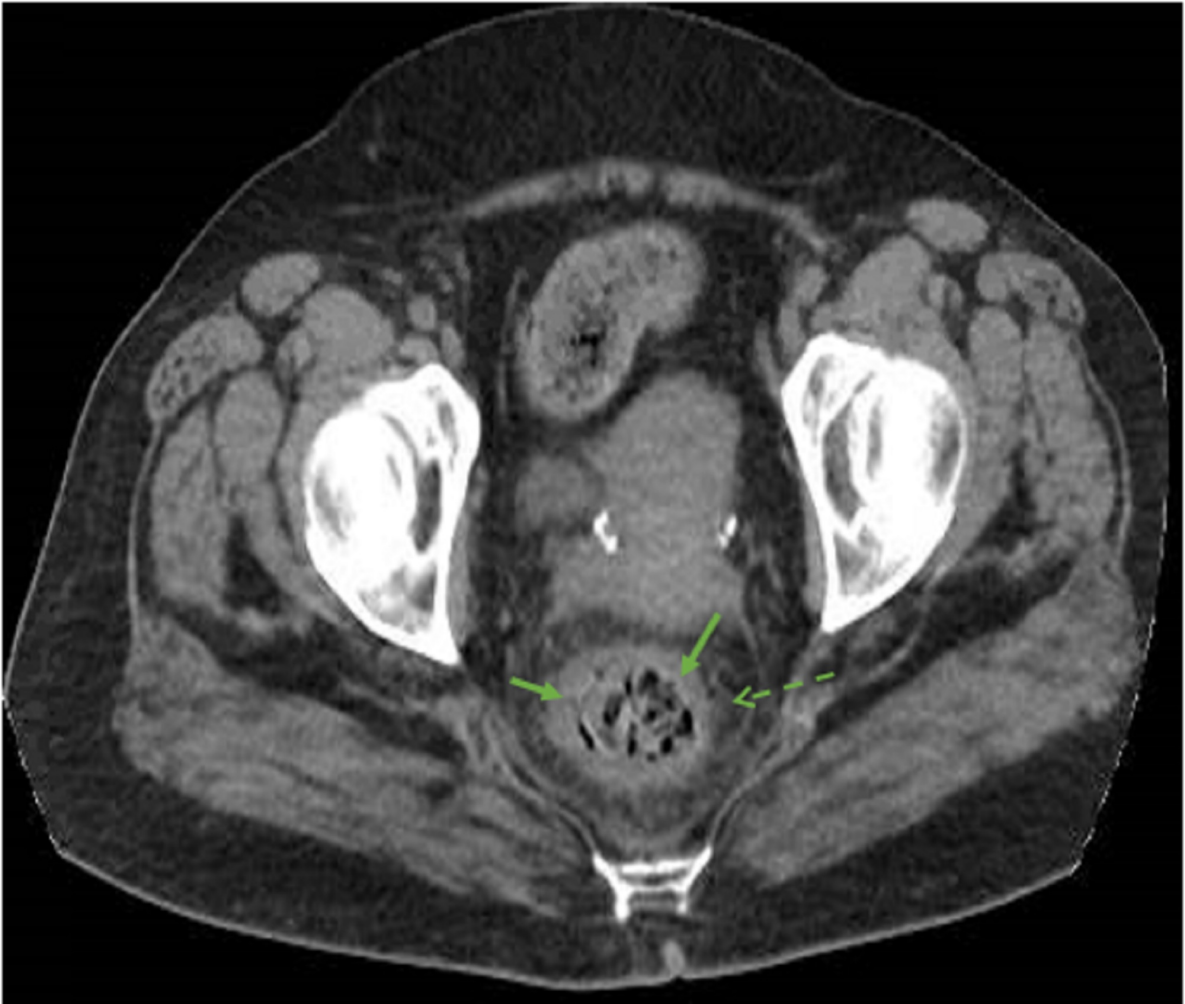
Inflammatory changes of the rectum compatible with proctitis. CT scan of the abdomen with intravenous contrast at the level of the rectum showing a circumferential wall thickening (full arrows) with fat stranding (dashed arrows) consistent with proctitis.

A 65-year-old female with a past medical history of diabetes mellitus, hypertension and psoriatic arthritis (on sulfasalazine 500 mg twice a day) presented to the emergency department after a syncopal episode. It was unwitnessed and she could not recall the event. She has been complaining of a mild generalized abdominal pain for one day and had a fever of 101°F prior to presentation. She was complaining of multiple mouth ulcers of new onset, causing dysphagia. On presentation, her blood pressure was 90/52 mmHg and heart rate 107 beats/min. The rest of the vital signs were normal, and she was afebrile. Physical exam was remarkable for oral ulcers and peri-umbilical tenderness on deep palpation. Initial blood work revealed severe neutropenia (white blood cell count (WBC) of 650/µL, absolute neutrophil count (ANC) of 60/µL), acute kidney injury (creatinine of 2.2 mg/dL and glomerular filtration rate [GFR] 23 mL/min) and a lactic acid of 3.9 mmol/L. She also had elevated inflammatory serum markers: erythrocyte sedimentation rate (ESR) 68 mm/hour (normal <40 mm/hour) and C-reactive protein (CRP) 3.10 mg/dL (normal <0.4 mg/dL). Stool lactoferrin was 31.50 µg/g (normal <7.24 µg/g) and calprotectin 231 µg/g (normal <50 µg/g). Her last blood work done one month ago had no abnormalities. CT scan of the abdomen showed evidence of colitis, involving the cecum, ascending colon and rectum, which in light of neutropenia was consistent with NE (Figures 1, 2).

Intravenous fluids and broad-spectrum antibiotics (cefepime, vancomycin and metronidazole) were initiated, and sulfasalazine was discontinued. She was admitted to the intensive care unit for neutropenic sepsis. Blood cultures were negative. She tolerated a diet; her pain was gradually resolving and she was subsequently downgraded to a medical floor.

On day 3 of hospitalization, the patient was clinically stable. Her blood work revealed a resolution of neutropenia with WBC 4,950/µL and ANC of 1,520/µL (Table 1). She was discharged home two days later with a full recovery of her neutrophil count and a complete resolution of her abdominal pain.

**Table 1:** Lab parameters on admission and three days after sulfasalazine discontinuation. WBC: white blood cell count; ANC: absolute neutrophil count ; BUN: blood urea nitrogen

	Day 1	Day 3
WBC (/µL)	650	4,950
ANC (/µL)	60	1,520
Lactic acid (mmol/L)	3.9	0.8
BUN (mg/dL)	31	12
Creatinine (mg/dL)	2.2	0.6

## Discussion

Even though NE is primarily described in the setting of malignancies and chemotherapy, one should keep in mind that this entity can occur in patients on any immunosuppressive therapy. It has been reported to occur in various immunosuppressed hosts, such as in AIDS, cyclic neutropenia and renal transplant patients [4-6]. In a systematic review regarding agranulocytosis induced by nonchemotherapy drugs, sulfasalazine was among the most common drugs responsible for neutropenia [7]. Bibbo et al. reported a case of typhlitis occurring in a patient treated with nafcillin for osteomyelitis [8]. It has also been described in a patient on maintenance methotrexate for rheumatoid arthritis [9].

It is still not well understood how NE develops. It is believed that there should be a mucosal injury to the colonic wall (most likely from a drug) and the host should be immunosuppressed [10]. Once those conditions co-exist and the gut barrier function has been lost, there will be a translocation of the gut bacteria subsequently starting a localized intestinal infection [11]. The cecum is most commonly involved since it has decreased vascularity and is rich in lymphatic tissue which makes it an optimal area for bacterial proliferation [12].

Sulfasalazine belongs to the family of the aminosalicylates and is metabolized into sulfapyridine and mesalamine (5-aminosalicylic acid or 5-ASA) via colonic intestinal flora [13]. It needs at least four weeks to start having an effect on the colonic mucosa and in order to do so, it needs to undergo acetylation in the blood [14]. Our patient was started on sulfasalazine eight weeks prior to presentation and eventually developed NE. Ullery et al. reported a case of NE presenting as an acute appendicitis which was successfully treated with antibiotics; no surgery was required. The patient was also started on sulfasalazine eight weeks prior to the presentation for a seronegative large joint arthritis [15].

Our patient most likely developed neutropenia secondary to sulfasalazine initially. She started to complain of oral mucositis (which is common in neutropenia and also seen in immunosuppressed patients undergoing chemotherapy). She then subsequently had a mucosal wall injury, which precipitated the colitis and proctitis. Early discontinuation of sulfasalazine and rapid initiation of antibiotic therapy were essential in the treatment of NE in this case. Current recommendations for lab monitoring in patients on sulfasalazine include a complete blood count (CBC) prior to therapy, then every other week for the first three months, then every month for the second three months, and then once every three months thereafter. Our patient last blood work was one month before presentation. She was supposed to have a more frequent monitoring of her CBC.

## Conclusions

The diagnosis of medication-related adverse reactions remains a big challenge for clinicians, and therefore requires a high index of suspicion. Resolution of the symptoms can simply occur with the discontinuation of the offending drug and often does not require extensive workup or treatments that might cause harm to the patient’s health. 
